# Cognitive and Physical Demands of Activities of Daily Living In Older Adults: Validation of Expert Panel Ratings: The SAGES Functional Measures Working Group

**DOI:** 10.1016/j.pmrj.2015.01.018

**Published:** 2015-02-04

**Authors:** Tamara G. Fong, Lauren J. Gleason, Bonnie Wong, Daniel Habtemariam, Richard N. Jones, Eva M. Schmitt, Sophia E. de Rooij, Jane S. Saczynski, Alden L. Gross, Jonathan F. Bean, Cynthia J. Brown, Donna M. Fick, Ann L. Gruber-Baldini, Margaret O’Connor, Patrica A. Tabloski, Edward R. Marcantonio, Sharon K. Inouye

**Affiliations:** 1 Aging Brain Center, Institute for Aging Research, Hebrew SeniorLife, Boston, MA; 2 Department of Neurology, Beth Israel Deaconess Medical Center, Harvard Medical School, Boston, MA; 3 Department of Medicine, Beth Israel Deaconess Medical Center, Harvard Medical School, Boston, MA; 4 Department of Geriatrics, Academic Medical Center, University of Amsterdam, Amsterdam, The Netherlands; 5 University of Massachusetts Medical School and Meyers Primary Care Institute, Worcester, MA; 6 Spaulding Rehabilitation Hospital, Boston, MA; Department of Physical Medicine and Rehabilitation, Harvard Medical School, Boston, MA; 7 Birmingham/Atlanta VA GRECC and School of Medicine, University of Alabama at Birmingham, Birmingham, AL; 8 School of Nursing, The Pennsylvania State University, Baltimore, MD; 9 Department of Epidemiology and Public Health, University of Maryland School of Medicine, Baltimore, MD; 10 William F. Connell School of Nursing, Boston College, Boston, MA

**Keywords:** Activities of Daily Living, expert panel, cognitive impairment, functional assessment, aging

## Abstract

**Background:**

Difficulties with performance of functional activities may result from cognitive and/or physical impairments. To date, there has not been a clear delineation of the physical and cognitive demands of activities of daily living.

**Objectives:**

To quantify the relative physical and cognitive demands required to complete typical functional activities in older adults.

**Design:**

Expert panel survey.

**Setting:**

Web-based platform.

**Participants:**

Eleven experts from eight academic medical centers and 300 community dwelling elderly adults age 70 and older scheduled for elective non-cardiac surgery from two academic medical centers.

**Methods:**

Sum scores of expert ratings were calculated and then validated against objective data collected from a prospective longitudinal study.

**Main Outcome Measurements:**

Correlation between expert ratings and objective neuropsychological tests (memory, language, complex attention) and physical measures (gait speed and grip strength) for performance-based tasks.

**Results:**

Managing money, self-administering medications, using the telephone, and preparing meals were rated as requiring significantly more cognitive demand, while walking and transferring, moderately strenuous activities, and climbing stairs were assessed as more physically demanding. Largely cognitive activities correlated with objective neuropsychological performance (r=0.13–0.23, *p*<.05) and largely physical activities correlated with physical performance (r=0.15–0.46, *p*<.05).

**Conclusions:**

Quantifying the degree of cognitive and/or physical demand for completing a specific task adds an additional dimension to standard measures of functional assessment. This additional information may significantly influence decisions about rehabilitation, post-acute care needs, treatment plans, and caregiver education.

## INTRODUCTION

Accurate and objective assessment of functional capacities is important in both clinical and research settings. Functional status is a core component of establishing clinical diagnoses, such as with dementia diagnosed using the Diagnostic and Statistical Manual of Mental Disorders-Fourth Edition (DSM-IV)^1^ or the National Institute on Aging-Alzheimer’s Association^2^ criteria, and is critical for determining capacity for independent living and need for assistive services. Moreover, objective assessments are necessary for capturing and tracking functional status over time, and contribute to the identification of post-acute care needs, and influences decisions about rehabilitation, treatment plans, and caregiver education.

Functional status is often measured through assessment of activities of daily living (ADLs). ADLs can be divided into two types: basic (BADLs), which comprise self-care tasks such as bathing, toileting, dressing, and grooming, and instrumental (IADLs), which include tasks that facilitate autonomy and independent living, such as handling finances, administering medications, shopping, and managing transportation.^3–5^ While BADLs are often thought to reflect more rudimentary, primarily physical, aspects of functioning and IADLs the more demanding cognitive aspects^6–8^ there is considerable overlap, as the ability to complete an activity can require highly divergent skills. For example, consider an individual who reports difficulty with shopping: this may reflect cognitive dysfunction (e.g., an inability to recall items needed, difficulty writing or retrieving the words to generate a list, finding the needed items or paying for them), physical impediment (e.g., difficulty walking to a store, pushing a cart down the aisles, carrying the groceries), or a combination of the two.

Understanding the relative contributions of cognitive or physical ability for successful completion of a task can have profound implications for determining optimal function and managing disability. For example, while a motorized scooter would help with mobility, such an adaptation could be useless or even dangerous if cognitive impairments preclude safe operation of this assistive device. Thus, quantifying the cognitive and physical contributions needed for completing an activity could yield more accurate information for predicting independence, detecting early functional decline, addressing patient safety, and tailoring treatment From a research perspective, knowing the differential impact of cognitive and physical ability to functional measures may reflect different underlying disease mechanisms and require different targeted intervention approaches focusing more on either physical or cognitive domains.

In this study, we convened a broad panel of multidisciplinary health care experts with limited representation of rehabilitation specialties who rated the relative cognitive and physical contributions to 16 activities taken from standard questionnaires of functional status. The expert ratings were used to assess the cognitive and functional contributions to these activities. To evaluate the validity of the ratings assigned by the experts, self-and proxy-reported data on these functional measures were evaluated in a cohort of community dwelling older adults awaiting major elective surgery, and compared with performance on neuropsychological testing and physical performance measures.

## METHODS

### Overall Study Design

The study team selected tasks for analysis (from ADLS, IADLS, MOS SF-12), the expert panel (n=11) was assembled, expert panel ratings were completed using a web-based visual analog rating scale, and ratings were validated against performance based tasks within a cohort sample (n=300).

### Selection of Functional Measures

The study team selected items requiring cognitive and/or physical skills from standardized questionnaires that measure functional activity and health status, including BADLs, IADLs, and the Medical Outcomes Study Short Form 12-item questionnaire (MOS SF-12).^4,9^ Items included from IADLs included managing money, administering medications, shopping, using transportation, preparing meals, using telephone, and doing housework. Items included from BADLs included dressing, bathing, grooming, feeding, toileting, transferring, and walking. Items included from the MOS SF-12 were doing moderate activities and climbing stairs; items that did not expressly measure a distinct activity or that asked about emotional or social roles and activities were not included. A total of 16 items were selected for the final analysis (Table 1). A copy of the full questionnaire is included as an Appendix 1.

### Expert Panel Rating

Eleven healthcare experts each with greater than ten years of individual experience in assessment of older patients for cognitive, physical, and functional capacity necessary for independent living, including a cognitive neurologist (TGF), two neuropsychologists (BW, MOC), three geriatricians (SKI, ERM, SD), a geriatrician and physical therapist (CB), two nurses (DF, PT), a physiatrist (JB) and a gerontologist (AGB) comprised the volunteer expert panel. The expert panel was selected based on nominations from faculty participating in the study. All panel members participated on a voluntary basis, and were chosen to represent broad interdisciplinary expertise across clinical and cognitive domains, although with limited representation of rehabilitation specialties (i.e. physiatry and physical therapy). A web-based visual analog rating scale (range 0 to 100, higher numbers reflecting more demand) was used to collect expert ratings. Experts were asked to independently and anonymously rate without discussion with other panel members the: (1) degree of cognitive demand, and (2) degree of physical demand of the selected activities and (3) the extent to which the activity is essential for independent daily living without regard to any specific study population. Experts were instructed to use their best professional judgment and provide ratings reflecting demand required for a typically healthy older adult, living independently in the community to manage each activity without assistance from another person and in an unaltered environment. The survey included two examples for panel members (1) consider an activity like jogging 5 miles-- one might rate this high on physical demand and relatively lower on cognitive demand, and low on essential for independent living; (2) consider another activity like fencing (as with foils)—one might rate this as high on physical demand and cognitive demand, but also low on essential for daily living.

### Data Collection

Ratings from the expert panel were averaged for each activity task included in the survey (Table 1). Items were then ranked by mean level of cognitive and physical contribution, such that each functional item could be categorized as being largely cognitive (high cognitive, low physical demand), largely physical (low cognitive, high physical demand) or a mixture of cognitive and physical (cognitive and physical rankings that fall near each other).

### Validation of Expert Ratings against Patient Data

Data from the Successful Aging after Elective Surgery (SAGES) study, an ongoing prospective observational study examining and following elderly patients scheduled for elective surgery described previously^10^, were used for validation of expert ratings following completion of expert survey. The cohort consisted of the three hundred patients (n=300) aged 70 years or older who were scheduled for elective major (non-cardiac) surgery at one of two acute care teaching hospitals in the Boston area. Exclusion criteria included active delirium or clinically recognized dementia. After enrollment in the study, patients completed a 75-minute interview in their homes prior to surgery with a standardized battery of assessments, including neuropsychological testing, physical functioning assessment, MOS SF-12^9^, and self and proxy-report of functioning on standard BADL and IADL instruments.^4^ Other key variables obtained included demographic information such as age, gender, marital status, and educational attainment, as well as a medical history. Interviews were also conducted with a proxy, who either lived with the patient or confirmed that they knew the patient well enough to report on his/her mental and physical abilities. The mean number of days between patient and proxy interviews was 3 days (standard deviation, SD, 8 days).

Neuropsychological measures in the SAGES study described in detail elsewhere included tests of memory, divided and sustained attention, working memory, and language.^10^ Overall cognitive function was determined by item response theory to generate a general cognitive performance measure^11^ using performance on the Trail-Making Tests, Parts A and B; Digit Span^12^; semantic fluency (FAS test)^13^; and the Hopkins Verbal Learning Test-Revised.^14^

Gait speed, grip strength, and the Minnesota Leisure Time Activities Questionnaire (MLTA)^15^ were used as proxy measures of physical performance. Gait speed was measured as the average of two 3-meter timed walking tests (meters/second). Grip strength (kilograms) was measured as the average of two trials where the participant was asked to grasp a hand dynamometer as tightly as possible. The MLTA provides an estimate of caloric expenditure from self-reported leisure activities, measured in kilocalories per week.

### Data Analysis

Standard descriptive statistics (means, standard deviations, proportions, and percentages) were used to describe the study sample and functional measure ratings. Expert ratings were normalized for each rater to account for the diverse ranges of scores used by different experts. Average normalized ratings were computed for each activity and served as weights for those activities. A weighted sum of scores on those activities with an average overall expert panel cognitive rating at least 1.5 times greater than their respective physical rating was calculated for each participant in the cohort (“Sum of Cognitive Items”) to indicate a large difference. Likewise, a weighted sum of scores for physical items at least 1.5 times greater than cognitive rating was created (“Sum of Physical Items”). The correlation of these sum scores with objective measures of neuropsychological (Trail-making tests, semantic fluency, HVLT-R delayed recall) and physical performance (gait speed, grip strength, MLTA) was examined, and Pearson correlation coefficients were obtained. A sum score (range 0–4, with a higher value indicating higher functioning) was created for cognitively (or physically) demanding items for each SAGES participant by totaling the number of cognitively (or physically) demanding activities he/she could perform.

### Human Subjects Approval

The Institutional Review Board (IRB) classified this study as exempt research and granted a waiver of informed consent for the expert panel members’ participation in the survey portion of this study. Panel members were informed prior to participation that the survey was anonymous and confidential, that no personally identifying information would be collected, and that answering any or all questions was entirely voluntary.

For the SAGES study, written informed consent was obtained from all participants, according to procedures approved by the IRB.

## RESULTS

### Panel Ratings of Cognitive and Physical Demand

**Figure 1:**
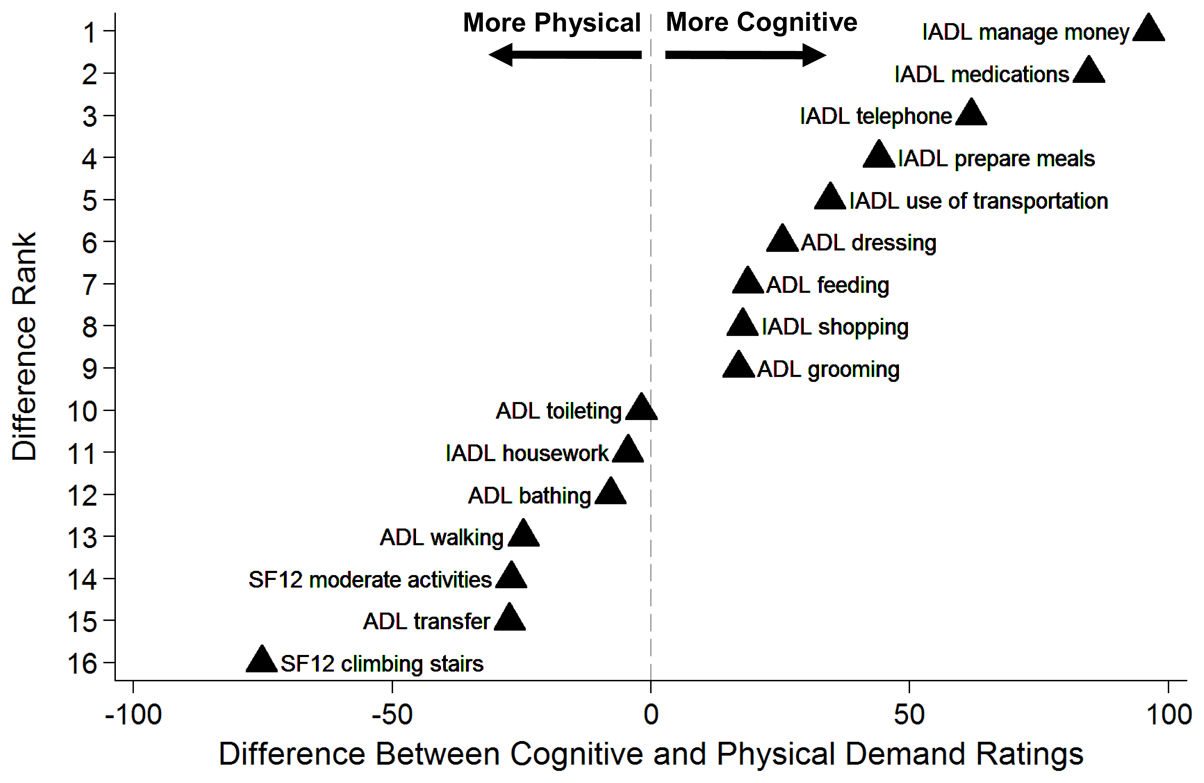
Difference in Mean Cognitive Ratings and Mean Physical Ratings By Expert Panel Difference scores between mean cognitive and mean physical ratings of 16 items by the expert panel is shown graphically. Positive values indicate greater cognitive than physical demand; negative values indicate more physical than cognitive demand. More Cognitive Items: IADL managing money, IADL medications, IADL telephone, IADL prepare meals; More Physical Items: SF12 climbing stairs, ADL transfer, SF12 moderate activities, ADL walking ADL = basic Activities of Daily Living; IADL = Instrumental Activities of Daily Living; MOS SF-12 = Medical Outcomes Study 12-item Short Form Survey Questionnaire; SD = standard deviation

Each of the sixteen selected activities scores from the BADL, IADL, and MOS SF-12 scales, rated on a 0–100 point scale for cognitive and physical demand (100 indicating highest demand) by the expert panel were averaged across all expert raters, and summarized in Table 1. Larger values indicated greater cognitive (or physical) contribution to the activity. Managing money, an IADL task, was assessed by panel members as highly cognitively demanding (97±7 points, mean ± standard deviation) but not physically demanding (1± 2 points), whereas climbing stairs (MOS SF-12) was rated as more physically demanding (96 ± 6 points) and less cognitively demanding (20 ±16 points) item. The scores were normalized across the raters. There was some lack of consensus for experts rating of items, as indicated by the different standard deviations for each item. Difference scores (i.e., the difference between mean cognitive and mean physical ratings) for each item are depicted graphically in Figure 1, displayed according to the rank of the difference scores. Items defined as cognitively demanding included managing money, managing medications, using the telephone, and preparing meals, whereas physically demanding items were walking, transferring, and MOS SF-12 items of managing moderate activities and climbing stairs.

### SAGES Cohort Characteristics

The SAGES cohort on average was 76.9 ± 5.0 years old, and the majority were white (95%), married (63%), and female (55%). On average, patients had 15.0 ± 2.9 years of education, and had only mild impairments on functional scales at baseline. Thus, the cohort represents a highly educated, generally healthy group of community-dwelling older individuals with little or no cognitive or functional impairment prior to surgery (Table 2).

### Validity of the Panel Ratings

To test convergent and discriminant validity of the expert panel ratings, items that were more heavily weighted for higher cognitive (or physical) demand were tested against objective measures of cognitive and physical function (Table 3). Both self-report and proxy-report functional measures were examined. For self-report functional measures, the sum score of the most cognitively demanding items correlated with all of the neuropsychological tests. Of the statistically significant correlations, the strongest of the correlations was with the general cognitive performance measure (r=0.23, *p*<.001), followed by Trails A (r= −0.18, *p*<.001) and Trails B(r =−0.17, *p*=.003). A sum score of the most physically demanding items was more strongly correlated with the physical measures, the MLTA (r=0.35, *p*<.001), gait speed (r=0.46, *p*<.001), and grip strength (r=0.15, *p*=0.23), than with neuropsychological test performance. Trails A and B correlated with both cognitive and physically demanding sum scores. For the proxy-report cognitive items (Table 4), the same relationships were found among the neuropsychological tests, except the correlation with delayed recall was not significant. For proxy-report functional measures, there was a significant correlation between the physically demanding sum score and physical performance measures, with the exception of grip strength.

## DISCUSSION

In this study, we identified that the IADL tasks of managing money, managing medications, using the telephone, and preparing meals were largely more cognitively than physically demanding. In contrast, ADL items of walking and transferring, and the MOS SF-12 items of moderate physical activities and climbing stairs were largely more physically than cognitively demanding. Some items, such as managing housework and toileting, had relatively similar cognitive and physical contributions. This study is innovative in its approaches to: (1) quantify and compare the relative contributions of cognitive and physical ability to measures of daily functioning; (2) draw upon a multidisciplinary panel of experts to devise quantitative ratings of the cognitive and physical demands of daily activities; and (3) validate expert ratings with respect to objective cognitive and physical tests.

In the current study, both self- and proxy-reported measures of functioning on the most cognitively demanding ADLs correlated most strongly with neuropsychological test scores, and self- and proxy-reported performance on physically demanding functional tasks correlated most strongly with objective physical performance tests, with a few exceptions. While there was significant correlation between performance on the HVLT-R Delayed Recall task and self-reported functioning on cognitively demanding ADLs, there was no correlation with delayed recall and proxy-report of cognitively demanding ADLs. This discrepancy may reflect the high-functioning nature of our study cohort, in which cognitive impairment, if present, may have been subtle and not readily apparent to proxies. This finding is consistent with that of prior studies where the correlation between cognitive performance and functional ability has been demonstrated to depend on whether reports of functioning originated with the patient or the proxy.^16–18^

Performance on Trail Making Tests correlated with self-reported performance on the most physically demanding ADLs (e.g., climbing stairs and transferring), which is not unexpected given the cognitive and physical demands (e.g. include using a pencil/pen to connect the letters and numbers), of the Trail Making Tests. However, the correlation was not seen with proxy-reports of performance. Likewise, grip strength correlated with the self- but not proxy-reported physically demanding functional measures. It is possible that grip strength is a small or indirect component of the most physically demanding items selected in this study. Again, the lack of correlation with the proxy-measures may reflect subtle functional deficits going unobserved by proxies.

In cases where measurement of cognitive and physical performance may not be feasible such as due to staffing or time constraints, functional status measures may be able to serve as surrogates to a limited degree. For example, inquiring about a cognitively demanding functional item, such as managing money, might be useful as part of an annual wellness screening for healthy elders, and further, impairment in a cognitively demanding functional item in the presence of subtle subjective cognitive complaints might represent an ultra-sensitive method for detecting early cognitive decline. Successful rehabilitation requires the cognitive skills associated with learning such as the ability to attend and retain new information. Identifying someone with difficulty on tasks associated with cognitive function might indicate a need for a longer course of rehabilitation or environment modification. Thus, early detection of cognitive decline through difficulty with functional tasks provides practical information that could help in the formulation of care plans and goals along with the medical recommendations.

Distinguishing between cognitively and physically demanding ADLs can provide important clinical insights into the basis for an individual’s impairment in daily functioning. Returning to the example of a patient reporting difficulty shopping, this ADL is not heavily weighted toward either end of the cognitive or physical spectrum (Figure 1). This would inform a clinician of the need for further investigation to identify a primarily cognitive or physical deficit, or both. Making this distinction may have ramifications for planning on appropriate post-acute discharge location (i.e. home vs. rehabilitation center) and/or the choice of targeted therapeutic regimes such as physical therapy for mobility or strength issues or occupational therapy for cognitive retraining or activities of daily living.

There are some limitations to this study. First, the cohort examined in the current study is both physically and cognitively high functioning; thus, generalizability of correlations between expert ratings and selected tasks to all elderly populations may be limited. In addition, given that the study participants were high-functioning and independent at baseline, proxy reporters may have had limited opportunity to observe subtle or early deficits. Thus, proxy reports may have had limited usefulness in this study and it is possible that additional or stronger correlations would have been found in a more impaired population. Both these issues represent important areas for future investigation. Lastly, while experts in physical therapy, cognitive and physical rehabilitation, and dementia care were included, our expert panel did not include professionals from occupational and speech therapy, given feasibility constraints and the voluntary nature of this study. These disciplines could have added a unique perspective as part of a multidisciplinary team. It is also possible that perspectives of professionals with similar training might differ from those of our expert panel and we included many experienced professionals from different institutions and locations to minimize this difference. Notably, the ratings from our expert panel had variable standard deviations for many items, suggesting that the panel represents a wide spectrum of opinions. These will be important considerations in future research expanding this work.

## CONCLUSIONS

By identifying the relative cognitive and physical demands of specific self- and proxy-reported functional tasks, a potential framework to predict performance on these tasks through the use of standardized functional assessments can be created. Future studies examining the correlation between expert ratings of the cognitive and physical demands of ADLs and actual daily functioning in other populations (e.g., patients with a broad range of common medical, cognitive and functional impairment) may provide important information for clinical care, education of caregivers and rehabilitation.

## Figures and Tables

**Table 1:** Expert Ratings on Selected Activities from Commonly Used Functional Measures^a^

Task	Cognitive Demand Rating, points (Mean ± SD)	Physical Demand Rating, points (Mean ± SD)
*IADL*		
Managing money	97 ± 7	1 ± 2
Administering medications	90 ± 15	5 ± 8
Shopping	84 ± 11	66 ± 16
Using transportation	79 ± 19	44 ± 25
Preparing meals	76 ± 20	31 ± 21
Using telephone	68 ± 25	6 ± 9
Doing housework	57 ± 24	62 ± 20
*ADL*		
Dressing	49 ± 18	23 ± 17
Bathing	44 ± 24	51 ± 25
Grooming	40 ± 26	23 ± 19
Feeding	25 ± 23	7 ± 5
Toileting	25 ± 16	27 ± 25
Transferring	8 ± 10	35 ± 29
Walking	5 ± 9	29 ± 18
*MOS SF-12*		
Doing moderate activities	42± 17	69± 22
Climbing stairs	20 ± 16	96 ± 6

a ADL = Basic Activities of Daily Living; IADL = Instrumental Activities of Daily Living; MOS SF-12 = Medical Outcomes Study 12-item Short Form Survey Questionnaire; SD = standard deviation.

Eleven multidisciplinary experts rated 16 activities on a visual analog scale (range 0 to 100, with higher values indicating higher demand) along two domains: degree of cognitive demand, and degree of physical demand. See text for details.

**Table 2:** Characteristics of the SAGES Participants, N = 300^a^

Characteristic	Mean ± SD a or n (%)
Demographic & Clinical Characteristics	
Age, years	76.9 ± 5.0
Male sex	134 (45)
Non-white/Hispanic	21 (7)
Married	170 (63)
Education, years	15.0 ± 2.9
Deyo-Charlson Comorbidity score	
None	88 (29)
One	69 (23)
Two or More	143 (48)
Planned surgery type	
Orthopedic	253 (85)
Vascular	16 (5)
Gastrointestinal	31 (10)
Functional Status Measures	
Any ADL impairment, self-reported	21 (7)
Any IADL impairment, self-reported	73 (24)
Any ADL impairment, proxy-reported	23 (8)
Any IADL impairment, proxy-reported	93 (32)
MOS SF-12 Physical subscore	35.6 ± 10.1
MOS SF-12 Mental subscore	50.2 ± 8.2
Cognitive Test Scores	
3MS score	93.2 ± 5.5
Neuropsychological Test Scores	
General Cognitive Performance summary score	57.2 ± 7.1
Trail Making Test, Part A, seconds	42.3 ± 15.4
Trail Making Test, Part B, seconds	115.9 ± 57.3
Semantic Fluency, number of words	21.3 ± 6.0
HVLT-R 20 minute delayed recall, number of words	7.3 ± 2.8
Physical Performance Measures	
MLTA, kcal/week	802.1 ± 949.5
Grip Strength, pounds	25.5 ± 10.3
Gait Speed, meters per second	0.7 ± 0.3
Sum scores created in this study^b^	
Cognitively Demanding Items, self-reported	3.9 ± 0.2
Physically Demanding Items, self-reported	2.9 ± 0.9
Cognitively Demanding Items, proxy-reported	3.9 ± 0.3
Physically Demanding Items, proxy-reported	2.9 ± 0.9

a 3MS = Modified Mini-Mental State examination; General Cognitive Performance summary score=performance based on factor analysis of the neuropsychological battery in SAGES; ADL = basic Activities of Daily Living; IADL = Instrumental Activities of Daily Living; HVLT-R = Hopkins Verbal Learning Test – Revised;MLTA = Minnesota Leisure Time Activity Questionnaire; MOS SF-12 = Medical Outcomes Study 12-item Short Form Survey Questionnaire; SD=standard deviation.

b A sum score was created for each participant by counting the number of cognitively (or physically) demanding activities he/she is able to do to, score 0–4, 4=high functioning. Cognitively demanding items included managing money, managing medications, shopping, and use of transportation; physically demanding items were walking and transferring, and MOS SF-12 items of managing moderate activities and climbing stairs.

**Table 3:** Validation of Self-Reported Cognitive and Physical Items, N=300^a^.

	Sum of Most Cognitively Demanding Items		Sum of Most Physically Demanding Items	
	*r*	*p*-value	*r*	*p*-value
	
*Neuropsychological Test Scores*				
General Cognitive Performance Factor	0.23	*<.001*	0.11	*ns*
Trail Making Test, Part A^b^	−0.18	*.001*	−0.12	*.033*
Trail Making Test, Part B^b^	−0.17	*.003*	−0.16	*.005*
Semantic Fluency	0.13	*.021*	0.02	*ns*
HVLT-R Delayed Recall	0.16	*.007*	0.10	*ns*
*Physical Performance Measures*				
MLTA	0.09	*ns*	0.35	*<.001*
Gait Speed	0.08	*ns*	0.46	*<.001*
Grip Strength	0.02	*ns*	0.15	*.023*

a HVLT-R = Hopkins Verbal Learning Test – Revised; MLTA = Minnesota Leisure Time Activities Questionnaire Score; ns = not significant; *r* = Pearson correlation coefficient. Cognitive Items selected were IADL managing money, managing medications, shopping, and use of transportation. Physical Items selected were walking and transferringand MOS SF-12 items of managing moderate activities and climbing stairs. Sum of Cognitive and Physical Items: score 0–4, 4=high functioning

b Trail Making Tests: higher scores reveal greater impairment.

**Table 4:** Validation of Proxy-Reported Cognitive and Physical Items, N=300^a^

	Sum of Most Cognitively Demanding Items		Sum of Most Physically Demanding Items	
	*r*	*p*-value	*r*	*p*-value
	
*Neuropsychological Test Scores*				
General Cognitive Performance Factor	0.19	*.001*	0.05	*ns*
Trail Making Test, Part A^b^	−0.23	*<.001*	−0.05	*ns*
Trail Making Test, Part B^b^	−0.15	*.013*	−0.09	*ns*
Semantic Fluency	0.13	*.021*	−0.02	*ns*
HVLT-R Delayed Recall	0.09	*ns*	0.04	*ns*
*Physical Performance Measures*				
MLTA	0.10	*ns*	0.36	*<.001*
Gait Speed	0.10	*ns*	0.47	*<.001*
Grip Strength	−0.02	*ns*	0.12	*ns*

a HVLT-R = Hopkins Verbal Learning Test – Revised; MLTA = Minnesota Leisure Time Activities Questionnaire Score; ns = not significant; *r* = Pearson correlation coefficient. Cognitive Items selected were IADL managing meals, managing medications, shopping and use of transportation. Physical Items selected were walking and transferring and MOS SF-12 items of managing moderate activities and climbing stairs. Sum of Cognitive and Physical Items: score 0–4, 4=high functioning

b Trail Making Tests: higher scores reveal greater impairment.

## Appendix 1

Dear Colleague,

We are asking you to make a rating, on a number of activities of daily living and physical performance tasks, (1) how physically demanding, (2) how cognitively demanding, and (3) the extent to which the activity is essential for independent living.

In making these ratings, please use your best professional judgment, and think about how demanding a task would be for a typically healthy older adult living independently in the community.

The rating scale is a visual analog scale (VAS: 0–100). Place your mouse over the tab on the bar, and slide it to the location that reflects your judgment. Note: to rate something as “0,” first slide the tab to the right and then back to the far left. You can go forward and backwards and change ratings. If you’d like to erase your setting and leave a response missing, press the reset button. Breathing is neither physically nor cognitively demanding but is essential for independent living.

EXAMPLE: Consider an activity like jogging 5 miles. One might rate this high on physical demand and relatively lower on cognitive demand, and low on essential for independent living. Consider another activity like fencing (as with foils). High on physical demand and cognitive demand, but also low on essential for daily living.

If you have any hesitancy in your ratings or would like to make a comment, please enter that in the comment block below the VAS.

When you are satisfied with your ratings, you can submit your scores using the submit button.

**Table:**

0-A task that is **not at all** physically demanding	100-A task that is **extremely** physically demanding
0-A task that is **not at all** cognitively demanding	100-A task that is **extremely** cognitively demanding

### Clinical Expert Survey

1. Bathing (includes having a sponge bath, tub bath, or shower)
2. Personal grooming, like brushing your hair, brushing your teeth, or washing your face
3. Dressing, like putting on a shirt, buttoning and zipping, or putting on shoes
4. Feeding like holding a fork, cutting food, or drinking from a glass
5. Getting from a bed to a chair
6. Using the toilet (like wiping yourself, or rearranging your clothes
7. Walking across a small room
8. Using the telephone
9. Getting to places out of walking distance
10. Going shopping for groceries
11. Preparing your own meals
12. Doing your own housework (household chores)
13. Managing your own money
14. Managing your own medications
15. Moderate activities, such as moving a table, pushing a vacuum cleaner, bowling, or playing golf
16. Climbing several flights of stairs
17. Your normal work, including work inside the home and housework
18. Walking for exercise
19. Moderately strenuous household chores like scrubbing, vacuuming
20. Moderately strenuous outdoor chores like mowing the lawn
21. Dancing
22. Bowling
23. Stretching or strengthening exercises
24. Squeezing dynamometer as hard as you can
25. Walking at a usual pace
